# Correction to: Robotic colorectal resection in combination with a multimodal enhanced recovery program - results of the first 100 cases

**DOI:** 10.1007/s00384-024-04611-0

**Published:** 2024-03-26

**Authors:** M. El-Ahmar, F. Peters, M. Green, M. Dietrich, M. Ristig, L. Moikow, J.-P. Ritz

**Affiliations:** 1 Department of general and visceral surgery, Helios Kliniken Schwerin, Wismarsche Straße 393 – 397, 19055 Schwerin, Germany; 2 Department of General and Visceral Surgery, Charité Universitätsmedizin Berlin Campus Benjamin Franklin, Hindenburgdamm 30, 12203 Berlin, Germany; 3 Department of Anesthesiology, Helios Kliniken Schwerin, Wismarsche Straße 393 – 397, 19055 Schwerin, Germany

**Correction to: International Journal of Colorectal Disease (2023) 38:95** 10.1007/s00384-023-04380-2

In this article additional affiliation has been added to the first author 'M. El-Ahmar' as 'Department of General and Visceral Surgery, Charité Universitätsmedizin Berlin Campus Benjamin Franklin, Hindenburgdamm 30, 12203 Berlin, Germany'.

